# Comparative Study of B Vitamins in Multiple Tissues of Oilseed Crops and Leafty Vegetables Reveal Sesame as a Valuable Resource in Vitamin B_3_, B_6_ and B_12_

**DOI:** 10.3390/antiox15020224

**Published:** 2026-02-09

**Authors:** Yijia Zhang, Ting Zhou, Zishu Luo, Desawi Hdru Teklu, Lei Wang, Rong Zhou, Wei Wang, Jun You, Huan Li, Linhai Wang

**Affiliations:** Key Laboratory of Biology and Genetic Improvement of Oil Crops of the Ministry of Agriculture and Rural Affairs, Oil Crops Research Institute of the Chinese Academy of Agricultural Sciences, Wuhan 430062, China

**Keywords:** edible plant tissues, dietary nutrition, sesame, antioxidant, plant-based sources

## Abstract

B vitamins are essential micronutrients for human health with prominent antioxidant properties, capable of scavenging reactive oxygen species (ROS) and maintaining redox homeostasis, protecting cells from oxidative damage. To address global nutrient deficiencies and identify plant-based antioxidant sources, this study quantified seven B vitamins (B_1_, B_2_, B_3_, B_5_, B_6_, B_9_, B_12_) in seeds, leaves, and seedlings of five oilseeds (sesame, peanut, soybean, rapeseed, perilla) and two leafy vegetables (spinach, lettuce) via LC-MS/MS, revealing distinct species- and tissue-specific patterns. Notably, sesame seeds exhibited exceptional vitamin B_3_ (niacin, 39.3 μg/g), surpassing other oilseeds1.6–8.2-fold; its leaves contained outstanding vitamin B_6_ (2.88 μg/g), 2.57–8.31-fold higher than spinach (1.12 μg/g) and lettuce (0.34 μg/g), and vitamin B_12_ (0.44 μg/g) levels ~13–20 times higher than other leaf samples. Sesame seedlings recorded high vitamin B_6_ (1.6 μg/g) and B_12_ (0.1 μg/g) among the oilseed crops seedlings. These findings highlight sesame as a multifunctional B vitamin resource for antioxidant nutrition, supporting dietary optimization, crop biofortification, and mitigation of global B vitamin inadequacies via plant-based solutions.

## 1. Introduction

B vitamins are a group of water-soluble micronutrients encompassing thiamine (vitamin B_1_), riboflavin (vitamin B_2_), niacin (vitamin B_3_), pantothenic acid (vitamin B_5_), pyridoxine (vitamin B_6_), biotin (vitamin B_7_), folate (vitamin B_9_), and cobalamin (vitamin B_12_), indispensable for sustaining redox balance and mitigating oxidative stress, in addition to their canonical roles in energy metabolism, neurotransmitter synthesis, and DNA repair [1,2,3,4,5,6]. Many B vitamins serve as core cofactors or precursors for the body’s antioxidant system: for example, vitamin B_2_ supports the activity of glutathione reductase, an enzyme that regenerates the key antioxidant glutathione (GSH) [7]; vitamin B_3_ is converted to NAD(P)H, which fuels ROS-scavenging enzymes [6]; and vitamin B_6_ (pyridoxine) regulates the metabolism of ROS-scavenging amino acids [3]. Deficiencies in these vitamins disrupt redox homeostasis, exacerbating oxidative stress and increasing risks of disorders such as pellagra—which is due to vitamin B_3_ deficiency, characterized by oxidative damage to skin and neurons [8]—and fetal neural tube defects arising from vitamin B_9_ or vitamin B_12_ deficiency, which are linked to oxidative DNA damage [9], as well as neurological impairments reinforced by vitamin B_1_ deficiency, which are associated with mitochondrial ROS overproduction [10]. These issues are globally prevalent, particularly in populations relying on plant-based diets lacking adequate antioxidant-rich B vitamin sources [8,10,11,12].

While animal products have long been recognized as primary sources of B vitamins, plant-based foods are gaining prominence amid rising vegetarian and flexitarian trends, offering sustainable natural antioxidant reservoirs [13,14]. Plant-based B vitamin contents vary substantially by species, genotype, and tissue type, with many oilseeds and leafy vegetables underexplored for their antioxidant-linked B vitamin potential [15,16]. For instance, black soybean seeds show significant intraspecific variation in vitamin B_6_ content [14], and maize displays broad variability in B vitamin content such as vitamin B_1_ (1.08–2.65 μg/g), vitamin B_3_ (0.2–2.14 μg/g), and vitamin B_6_ (1.4–10.65 μg/g) [17]. Existing studies have predominantly focused on crops, such as rice and wheat, or individual vitamins, with limited attention to comparative analyses across multiple oilseed crops and leafy vegetables or to the influence of tissue type (seeds, leaves, seedlings) and genotype factors on vitamin accumulation, limiting the development of natural antioxidant sources [15,16,18,19,20]. However, B_12_ from plant sources has a lower bioavailability than B_12_ from animal sources, because its absorption is impeded by dietary matrix components (e.g., phytates, fiber) and requires the secretion of sufficient intrinsic factors in the human gut [21]. Nowadays, the way is also being opened for the effective provision of B_12_ in plant foods through novel technologies such as the production of the B_12_ precursor betaine using recombinant Pseudomonas aeruginosa [22].

Notably, sesame, a crop valued for its oil content, has been overlooked for its micronutrient and antioxidant potential, despite preliminary evidence of its B vitamin richness [23,24,25]. This oversight is significant, as sesame is widely cultivated across diverse agro-climatic zones globally, making it a potential candidate for targeted biofortification for nutritional security. Given that oxidative stress underlies numerous chronic diseases and that over two billion people face micronutrient deficiencies worldwide [26], identifying plant-based sources with high, tissue-specific antioxidant B vitamin content is critical for advancing natural antioxidant research and dietary interventions. Hence, thisstudy extends existing LC-MS/MS-based B vitamin surveys through three key innovations: a multi-tissue comparative design that integrates seed, leaf, and seedling tissues across five oilseed crops and two leafy greens, moving beyond the seeds focus of most prior studies to reveal comprehensive tissue-specific B vitamin partitioning patterns; the inclusion of seedlings, a largely understudied edible tissue, to uncover B vitamin dynamics during early plant development and expand dietary application scenarios; and a large-scale analysis of sesame germplasms to characterize genetic variability in B3 content, which provides a foundation for future biofortification programs. These findings have the potential to contribute to the scientific basis for optimizing dietary antioxidant intake, developing functional foods and designing crop biofortification strategies targeting B vitamin enrichment.

## 2. Materials and Methods

### 2.1. Plant Materials, Growth Conditions and Sample Collection

Seeds (Sesame, soybean, peanut, rapeseed, perilla), leaves (Sesame, rapeseed, perilla, lettuce and spinach), seedlings (Sesame, rapeseed, peanut) were used in the present study. Materials and tissues were selected based on their edible application value. Seeds were sourced from the National Medium-Term oilseed crops Gene Bank of China (Wuhan, China). Whereas, the leafy vegetables were purchased from local markets. Sesame, soybean, peanut, rapeseed and perilla were grown under natural conditions at the experimental station of the Oil Crops Research Institute of Chinese Academy of Agricultural Sciences (OCRI-CAAS, Wuhan, China) during 2024. Each material was planted with three cultivars. Field management of the trials were followed the standard agronomic practices for each crop. The experiment was conducted in a randomized complete area design, sampling in a 3-row area with a compartment width of 1.8 m, compartment furrows of 0.4 m, row spacing of 0.4 m, and plant spacing of 0.2 m. In order to avoid edge effects, seeds were harvested from the middle 10 plants of each plot, and each replicate was mixed to form a composite sample, and each composite was analyzed in three technical replicates. Fresh leaves of sesame, perilla and rapeseed were sampled from three-weeks-old plants. Seeds of sesame, soybean, peanut, rapeseed and perilla were harvested at physiological maturity stage. One-week-old seedlings of soybean, peanut, and sesame were sampled for further experiments. The study was conducted following with the local and national plant regulations.

### 2.2. Reagents and Instruments

Analytical standards for vitamins B_1_ (thiamine) (CAS 67-03-8, China), B_2_ (riboflavin) (CAS 83-88-5, China), B_3_ (niacin) (CAS 59-67-6, China), B_5_ (pantothenic acid) (CAS 137-08-6, China), B_6_ (pyridoxine) (CAS 58-56-0, China), B_9_ (folic acid) (CAS 59-30-3, China), and B_12_ (cobalamin) (CAS 68-19-9, China)were purchased from Shanghai Yuanye Biotechnology (Shanghai, China). HPLC-grade acetonitrile and formic acid were acquired from Merck (Darmstadt, Germany), and analytical-grade hydrochloric acid and sodium hydroxide were supplied by Cologne Chemicals (Chengdu, China). Ultrapure water (18.2 MΩ·cm) was generated using a Milli-Q system. Instruments included a Shimadzu ATY224 electronic balance (Kyoto, Japan), KQ3200DA ultrasonic cleaner (Kunshan, China), H3-18KR high-speed centrifuge, Sartorius pH meter, and AB Sciex QTRAP 5500 LC-MS/MS system (Marlborough, MA, USA).

### 2.3. Sample Preparation and Extraction

Samples were frozen in liquid nitrogen for 30 min, and the frozen samples were transferred to a freeze dryer (model: Alpha 1-2 LDplus, Christ, Germany) at 0.08 mbar, −55 °C for 48 h until constant weight for B vitamin determination. For B vitamins extraction, 0.5 g of sample was accurately weighed into a centrifuge tube, mixed with 4.5 mL ultrapure water, vortexed for 30 s, and sonicated in an ice bath for 30 min. The extract pH was adjusted to 4.5 with 6 M hydrochloric acid, incubated for 2 min, and neutralized to pH 7.0 with 2 M sodium hydroxide. After centrifugation at 10,000× *g* for 10 min, the supernatant was diluted to 10 mL, filtered through a 0.22-μm PTFE membrane, and stored at −20 °C prior to analysis.

### 2.4. LC-MS/MS Analysis

Chromatographic separation was performed on a Waters XSelect® HSS T3 column (Milford, MA, USA) (4.6 mm × 250 mm, 3.5 μm) at 30 °C using a mobile phase of 0.2% formic acid in water and acetonitrile. The gradient elution program was 5% acetonitrile for 2 min, increasing to 20% over 8 min, 95% over 2 min, held for 1 min, and returned to 5% over 1 min, at a flow rate of 0.40 mL/min with a 2-μL injection volume.

Mass spectrometry was conducted in ESI+ mode with MRM detection. Source parameters included a curtain gas of 30 psi, +4500 V spray voltage, 500 °C temperature, and 50 psi for nebulizer and auxiliary gases. Quantitative analysis used the following parent/daughter ion pairs: VB1 (265.0→122.0), VB2 (377.0→243.2), VB3 (124.0→80.0), VB5 (220.0→202.0), VB6 (170.0→152.0), VB9 (442.0→295.2), VB12 (678.0→147.0), with optimized collision energies (REF). Calibration curves (1–1000 ng/mL) were prepared in 50% methanol-water, and quality control samples were analyzed every 10 injections to ensure method reproducibility.

### 2.5. Statistical Analysis

All the experiments were performed with three biological replicates, and the presented values are the mean ± SD. Data processing was performed using IBM SPSS Statistics 27. Statistical significance was determined using analysis of variance (ANOVA) with Duncan’s multiple range test (*p* < 0.05) and Student’s *t*-test (*, *p* < 0.05 is considered significantly different, and **, *p* < 0.01 is considered highly significantly different). Heat maps were generated by GraphPad Prism 8 (standardization via mean centering and standard deviation).

## 3. Results and Discussion

### 3.1. B Vitamin Profiles in Oilseed Crop Seeds

**Figure 1:**
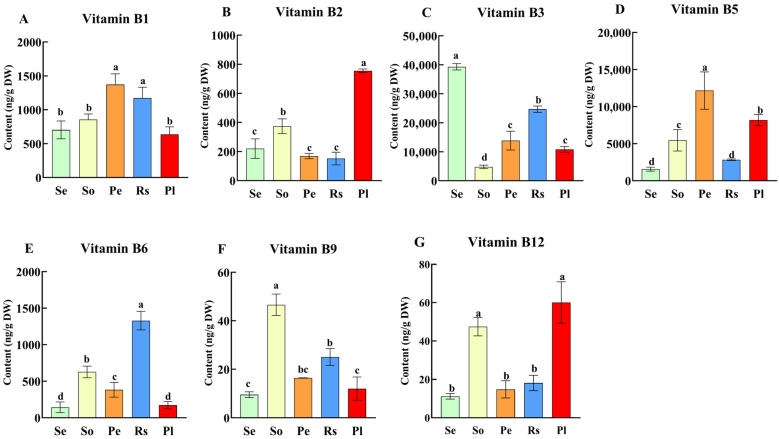
B vitamin variations [B_1_ (**A**), B_2_ (**B**), B_3_ (**C**), B_5_ (**D**), B_6_ (**E**), B_9_ (**F**), and B_12_ (**G**)] in seeds of five oilseed crops (Se: sesame, So: soybean, Pe: peanut, Rs: rapeseed, and Pl: perilla). Different letters indicate significant differences in vitamin B content among seeds of different crops (*P* < 0.05).

B vitamins are unable to be synthesized and stored by humans, and these micronutrients are essential for their daily diet [12]. Plants or plant-based foods are good sources of B vitamins, with natural variations in their contents contributing to diverse nutritional applications [16]. In the present study we detected the seven types of B vitamin complexes (vitamin B_1_, B_2_, B_3_, B_5_, B_6_, B_9_, and B_12_) in seeds of five oilseed crops such as sesame, soybean, peanut, rapeseed and perilla. Quantitative analysis revealed significant interspecific differences in B vitamin profiles across the five oilseed crops seeds (Figure 1). Sesame seeds exhibited the highest content of vitamin B_3_ (39.3 μg/g), 1.6-fold levels higher than rapeseed (24.7 μg/g), 2.8-fold higher than peanut (13.8 μg/g), 3.6-fold higher than perilla (10.8 μg/g) and 8.2-fold higher than soybean (4.78 μg/g). This exceptional vitamin B_3_ content not only positions sesame seeds as a superior natural functional ingredient for niacin supplementation but also their potential as a source of antioxidant-related nutrients, supported by their biochemical role. Specifically, vitamin B_3_ plays a critical role in maintaining cellular redox homeostasis by participating in the synthesis of nicotinamide adenine dinucleotide (NAD+) and nicotinamide adenine dinucleotide phosphate (NADP+), key cofactors involved in antioxidant defense and ROS scavenging [27]. The high content of B_3_ available in sesame seeds makes the crop ideal for incorporation into processed foods like bread, snacks, and plant-based meat alternatives to simultaneously enhance nutritional value and antioxidant capacity. Additionally, the higher sesame seeds’ B_3_ concentration in the present study exceeds that of major cereal crops: for instance, it is 22.9–245-fold higher than the bioavailable B_3_ in wheat (0.16–1.74 μg/g dw) [28] and 18.4–200-fold higher than the B_3_ range in maize (0.196–2.1375 μg/g) [17], highlighting sesame’s unique potential to enhance niacin intake, being particularly valuable for preventing pellagra, a B_3_ deficiency disorder, in regions reliant on cereal-dominated diets [8].

Among other oilseeds, peanut seeds revealed the highest vitamin B_1_ and vitamin B_5_ contents, with a value of 1.37 μg/g and 12.2 μg/g, respectively. These findings could benefit regions with rice-dominant diets, where thiamine (vitamin B_1_) deficiency contributes to beriberi disease, an effect potentially reinforced by vitamin B_1_’s role in protecting mitochondrial function against oxidative damage [29]. Moreover, rapeseed showed the highest vitamin B_6_, with a content of 1.33 μg/g. The vitamin B_9_ content in soybean (0.046 μg/g) was significantly higher compared to other crops. Perilla was recorded with higher in vitamin B_2_ (0.75 μg/g) and vitamin B_12_ (0.06 μg/g) contents than other crops. Among the seven studied B vitamin complex, vitamin B_3_ content was highly available in the five oilseed crops, ranging between 4.78 and 39.3 μg/g, emphasizing oilseeds as a collectively valuable resource for both nutritional supplementation and antioxidant defense. These variations indicated that different oilseeds might serve as complementary sources to meet specific B vitamin and antioxidant-related nutrition, diversifying seed-based nutritional strategies.

### 3.2. B Vitamin Profiles in Oilseed Crop Leaves and Leafy Vegetable Leaves

**Figure 2:**
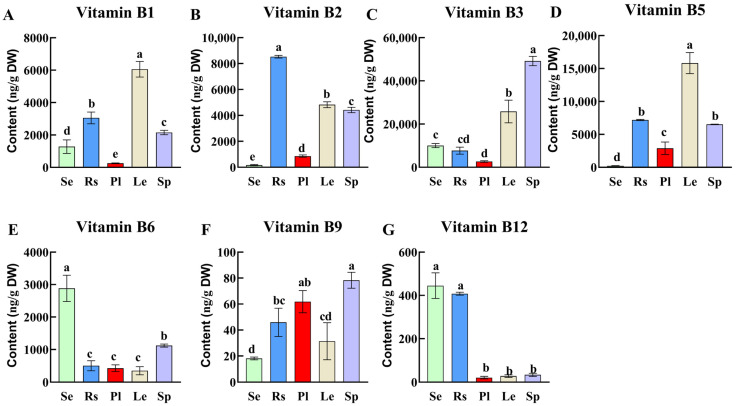
B vitamin variations [B_1_ (**A**), B_2_ (**B**), B_3_ (**C**), B_5_ (**D**), B_6_ (**E**), B_9_ (**F**), and B_12_ (**G**)] in leaves of three oilseed crops (Se: sesame, Rs: rapeseed, and Pl: perilla) and leafy vegetables (Le: lettuce and Sp: spinach). Different letters indicate significant differences in B vitamin content among plant leaves (*p* < 0.05).

Based on quality and high preference for cooking oil production, we selected three edible oilseed crops, including sesame, rapeseed and perilla leaves, and two regularly consumed leafy vegetables (spinach and lettuce) to determine the B vitamin variations in the study (Figure 2). Of the leave samples, lettuce showed the highest vitamin B_1_ (6.05 μg/g) and vitamin B_5_ (15.83 μg/g). The highest vitamin B_2_, B_3_, and B_6_ values of 8.53 μg/g, 49.17 μg/g, and 2.88 μg/g were recorded in the leaves of rapeseed, spinach, and sesame, respectively. Similarly, higher values in vitamin B_9_ (0.08 μg/g) and B_12_ (0.44 μg/g) were recorded for spinach and sesame leaves, respectively.

Typically, the vitamin B_6_ content in sesame leaves was 2.57, 8.31, 5.76, and 6.8 times higher than in spinach, lettuce, rapeseed, and perilla, respectively. Vitamin B6 (pyridoxine) is a critical antioxidant cofactor that modulates the glutathione (GSH) system, an essential component of cellular antioxidant defense [30]. This function is particularly important because staple foods such as rice, wheat, and cassava often fail to meet recommended dietary intake of vitamin B6 when consumed as the primary source of nutrition. Given the known biochemical functions of vitamin B6, such dietary deficiencies might cellular antioxidant capacity and increase the risk of oxidative stress [3]. In contrast to the present finding, low B_6_ content (1.27–2.97 μg/g dw) reported by Shewry et al. [28] in wheat grains and sesame leaves could be a significant supplementary source for B_6_.

Moreover, sesame (0.44 μg/g) and rapeseed (0.4 μg/g) leaves contained the highest vitamin B_12_ levels, approximately 13-, 16-, and 20-fold higher than those of spinach, lettuce and perilla, respectively. Vitamin B_12_ is typically scarce in plant-based diets, and it exerts a key biochemical role in maintaining cellular redox homeostasis by lowering homocysteine levels—an imbalance of which is associated with elevated oxidative stress [31]. Notably, the B_12_ contents in sesame (0.44 μg/g) and rapeseed (0.4 μg/g) leaves exceed that of shiitake mushrooms, a plant source long recognized for its relatively high B_12_ content, with a reported range of 0.013 to 0.127 μg/g [20]. The relatively high and stable B_12_ concentration in sesame leaves addresses a critical nutritional gap in vegetarian diets, providing a reliable plant-based source of B_12_, which might help prevent megaloblastic anemia and support cellular redox balance, thereby reducing the risk of oxidative stress-induced DNA damage [31,32].

In contrast, lettuce leaves had approximately twice the B_1_ and B_5_ levels compared to sesame, rapeseed, perilla, and spinach. Collectively, these findings highlight the tissue-specific, distinct biochemical roles linked to cellular redox regulation, with sesame leaves emerging as a dual source of B6 and B12 for nutritional supplementation and the amelioration of redox imbalance associated with B vitamin deficiency—particularly valuable for vegetarians and populations at risk of oxidative damage related to inadequate B vitamin intake.

### 3.3. B Vitamin Profiles in Oilseed Crop Seedlings

**Figure 3:**
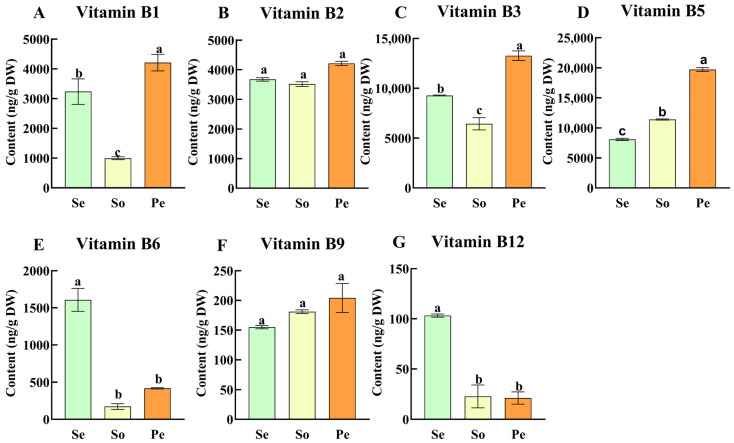
B vitamins variation [B_1_ (**A**), B_2_ (**B**), B_3_ (**C**), B_5_ (**D**), B_6_ (**E**), B9 (**F**), and B_12_ (**G**)] in seedlings of three oilseed crops (Se: sesame, So: soybean, and Pe: peanut). Different letters indicate significant differences in B vitamin content among seedlings of different crops (*p* < 0.05).

We also investigated the B vitamin variations in the seedlings of three edible oilseed crops, including sesame, peanut, and soybean (Figure 3). Results revealed that there were no significant differences in contents of vitamin B_2_ and B_9_, while remarkable variation occurred among the other five B vitamins (B_1_, B_3_, B_5_, B_6_, B_12_). Peanut seedlings were evidenced to be rich in vitamin B_1_ (4.21 μg/g), B_3_ (13.27 μg/g), and B_5_ (19.7 μg/g), while sesame seedlings followed with contents of 3.23 μg/g, 9.28 μg/g, and 8.08 μg/g, respectively. Sesame seedlings had stood out with the highest vitamin B_6_ (1.6 μg/g) and B_12_ (0.1 μg/g), reinforcing sesame’s multi-tissue potential as a source of antioxidant-related B vitamins.

Seedlings represent underutilized nutritional reservoirs, with their B vitamin profiles reflecting adaptations to early growth under oxidative stress. Vitamin B_6_ in sesame seedlings contributes to ROS scavenging and GSH maintenance [30], while B_12_ supports DNA integrity by reducing oxidative damage [31]. These properties make seedlings ideal for integration into urban farming and ready-to-eat products (e.g., salads, spreads), offering convenient sources of both nutrients and antioxidants for modern lifestyles. It should be noted, however, that plant-derived B12 exhibits inherently lower bioavailability than animal-derived forms, as its absorption is hindered by dietary matrix components (e.g., phytates, fiber) and requires sufficient intrinsic factor secretion in the human gut; additionally, both B6 and B12 are easily degraded under harsh food-processing and storage conditions (e.g., exposure to light, oxygen, extreme temperatures, and pH fluctuations), which may compromise their nutritional efficacy before consumption. The complementary B vitamin profiles of peanut, soybean, and sesame seedlings further enable diversified dietary strategies, where each crop’s seedlings can be leveraged to target specific antioxidant and nutritional needs [33].

### 3.4. Tissue-Specific Accumulation of B Vitamin in Different Oilseed Crops

**Figure 4:**
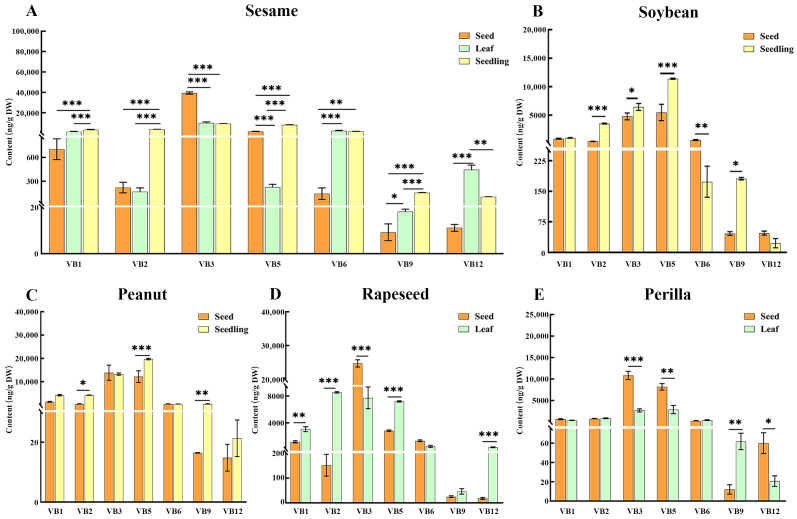
Comparison of B vitamin content among different tissues of five oilseed crops. Sesame (**A**), soybean (**B**), peanut (**C**), rapeseed (**D**), and perilla (**E**). *, **, *** represent *p* < 0.05, *p* < 0.01, and *p* < 0.001, respectively.

The vitamin B contents were compared between the seed and non-seed tissues (leaf, and seedling) in the five oilseed crops. Results revealed significant differences among the studied tissues of the oilseed crops in the present study, highlighting the need for comprehensive utilization of crop tissues for optimal B vitamin and antioxidant intake. Sesame exhibited the most pronounced tissue partitioning: seeds contained substantially higher B_3_ levels than leaves or seedlings, while leaves and seedlings had markedly elevated B_6_ and B_12_ concentrations relative to seeds (Figure 4A). This pattern aligns with functional roles of these vitamins: B_3_ (niacin) in seeds likely supports oxidative stress defense during long-term storage, whereas B_6_ and B_12_ in photosynthetic leaf tissues and actively growing seedlings are critical for ROS scavenging during photosynthesis and early development (Mangel et al., 2019) [34]. Additionally, vitamin B_12_ can influence the activity of superoxide dismutase (SOD) through direct or indirect mechanisms, thereby playing a central role in regulating the body’s redox homeostasis [35]. Studies indicate that vitamin B6 enhances ascorbic acid oxidase activity in coffee leaves, leading to increased chlorophyll content and improved photosynthetic capacity in plants. The mode of action of vitamin B6 in sesame leaves may be consistent with this mechanism [36]. Rapeseed leaves had 56 times higher vitamin B_2_ and 19 times higher vitamin B_12_ than seeds, while seeds had 3 times higher vitamin B_3_ than leaves (Figure 4B). Vitamin B2 (riboflavin) serves as a precursor for two crucial coenzymes—flavadenosine dinucleotide (FAD) and flavin mononucleotide (FMN)—which play vital roles in cellular energy metabolism and redox reactions. It reduces oxidative damage in plants by decreasing the levels of acetaldehyde, H_2_O_2_, hydroxyl radicals, and lipoxygenase activity. Rapeseed leaves may enhance their antioxidant activity through these mechanisms [37]. Perilla seeds had 4 times higher vitamin B_3_ and 2.5 times higher vitamin B_5_ than leaves, but vitamin B_9_ content in seeds was 5 times lower than that in leaves (Figure 4C). Soybean seedlings had 9 times higher vitamin B_2_, 1.3 times higher vitamin B_3_, and 3 times higher vitamin B_9_ than seeds (Figure 4D). Plant folic acid metabolism maintains REDOX balance by promoting the production of NADPH through a reaction catalyzed by methylenetetrahydrofolate dehydrogenase, enabling plants to cope with oxidative stress [38]. In peanuts, seedlings were significantly higher in vitamin B_5_ and vitamin B_9_ than seeds (Figure 4E). The results suggested the seeds of sesame and rapeseed are better resources for vitamin B_3_ than their leaves and seedlings; the leaves of sesame and rapeseed can supply more vitamin B_12_ than their seeds; Perilla seeds are good resources for vitamin B_3_ and vitamin B_5_ than its leaf; and rapeseed leaves and soybean seedlings are good resources for vitamin B_2_ than their seeds. The results revealed distinct tissue-specific patterns. These patterns challenge the traditional focus on seeds, emphasizing that non-seed tissues (leaves, seedlings) can be equally valuable for dietary B vitamin intake and antioxidant defense. Collectively, these findings support the comprehensive utilization of oilseed crops, where each tissue can be targeted for specific B vitamin and antioxidant applications.

### 3.5. B Vitamin Profiles in Black and White Sesame Seeds Across Tissues

**Figure 5:**
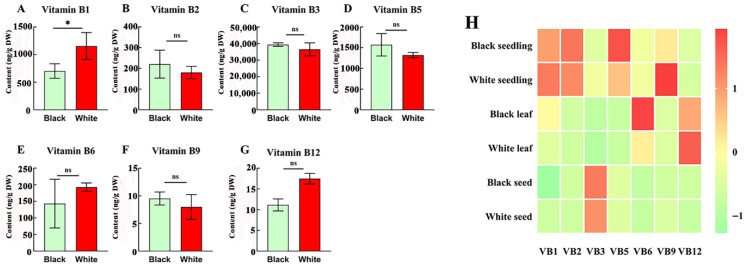
Comparison of the contents of B vitamins [B_1_ (**A**), B_2_ (**B**), B_3_ (**C**), B_5_ (**D**), B_6_ (**E**), B_9_ (**F**) and B_12_ (**G**)] in black and white sesame seeds. (**H**) Heat map of relative content of seven B vitamins in different tissues of black and white sesame seeds. * represents *p* < 0.05; ns represents no significant difference.

Black sesame seeds are usually considered to have high value for their antioxidative ability in the treatment of diverse diseases, while white seeds were valued in oil extraction [39]. Comparative analysis of black and white sesame seeds revealed tissue-specific B vitamin distributions, with seed coat color influencing only vitamin B_1_ content in seeds. White sesame seeds showed significant differences and had 1.5 times higher vitamin B_1_ content of 1.09 μg/g than black seeded sesame (0.7 μg/g) (Figure 5A), while there were no significant differences (ns) observed in the remaining B vitamins in the current study (Figure 5B–G). The heat map results showed both white and black sesame seedlings exhibited the highest vitamins B_1_ (3.23 and 2.36 μg/g,, respectively), B_2_, (3.67 and 3.65 μg/g, respectively), and B_5_ (8.1 and 10.5 μg/g, respectively), whereas seeds had peak vitamin B_3_ (35.5 and 39.3 μg/g, respectively). The leaves of both seed colors stood out with the highest vitamin B_12_ (0.44 and 0.48 μg/g respectively), but black sesame leaves showed 2.76–22-fold higher vitamin B_6_ levels than other two tissues (Figure 5H). These findings indicate that black sesame seeds may offer advantages for vitamin B_5_ in seedlings and vitamin B_6_ in leaves, supporting targeted utilization based on tissue and seed coat color.

### 3.6. Nutritional Evaluation of Colored Sesame Seeds: Implications for Targeted Dietary Utilization

Vitamin B_3_ (niacin) is a key nutrient that enhances cellular redox defense by promoting NAD(P)H-dependent ROS scavenging and inhibiting the biochemical process of lipid peroxidation [8]. To identify sesame germplasm with high vitamin B_3_ content and thereby expand vitamin B_3_ supply sources, we analyzed vitamin B_3_ variability across different sesame germplasms (Table 1). The results showed that vitamin B_3_ content in sesame germplasm exhibited substantial variability across the tested germplasms. Among 92 total samples, the content of vitamin B_3_ ranged from 23.57 to 82.25 μg/g, with a mean of 39.28 μg/g and a CV of 15.12%. White sesame showed a range of 23.57–55.87 μg/g, with a mean of 38.79 μg/g and a CV of 16.44%, while black sesame had a narrower distribution, ranging from 25.37 to 45.14 μg/g, with a mean of 38.7 μg/g and a CV of 13.93%. Notably, yellow sesame ranged from 26.24 to 82.25 μg/g, exhibiting the highest mean (40.66 μg/g) and greatest variability (CV = 32.83%, matching the overall maximum), whereas brown sesame displayed intermediate variation (24.08–48.64 μg/g; mean = 37.90 μg/g; CV = 18.94%). These results underscore the broad natural variation in vitamin B_3_ content across sesame germplasm, informing the screening of high-vitamin B_3_ accessions to boost vitamin B_3_ supply. This genetic diversity enables targeted breeding for high-B_3_ germplasms. The identification of germplasms with B_3_ contents enables the development of tailored fortification from daily staples to high-dose products and enhancing sesame’s role in addressing global niacin inadequacies and oxidative stress-related health issues.

### 3.7. Implications for Dietary Diversification, Biofortification and Antioxidant Defense

Globally, achieving a balanced and diverse diet remains a challenge for several people, attributable to micronutrient deficiencies, particularly in low-income settings [40]. A diversified diet implies the adequate intake of essential nutrients and antioxidants, and plant-based sources are increasingly recognized for their dual role in nutrition and oxidative stress mitigation [41]. Understanding the plant-based sources and profiles are essential for dietary diversification and fortification, contributing to nutrient deficiency globally. The distinct species- and tissue-specific B vitamin patterns highlight the potential of oilseeds, particularly sesame, as multifunctional resources. Sesame’s seeds (high B_3_), leaves (high B_6_, B_12_), and seedlings (high B_6_, B_12_) allow comprehensive dietary integration, from seed-based products to leafy greens and sprouts. These tissues collectively provide a spectrum of B vitamins, each of which may support antioxidant activity through pre-existing biochemical actions: B3 is involved in NAD(P)H synthesis, B6 for GSH maintenance, and B12 for homocysteine reduction [27,31]. Furthermore, sesame’s adaptability to diverse agro-climatic zones and compatibility with local diets in regions like South Asia and East Africa further enhance its utility as a sustainable solution for global nutritional and antioxidant needs.

Leveraging the observed variations, future efforts can focus on breeding high-B vitamin germplasms and promoting the inclusion of non-seed tissues in diets. These strategies hold promise for addressing global micronutrient deficiencies through sustainability. Additionally, integrating sesame into biofortification programs can enhance the antioxidant capacity of staple diets, reducing the risk of chronic diseases linked to oxidative stress (e.g., cardiovascular disease, neurological disorders) [1,4].

## 4. Conclusions

**Figure 6:**
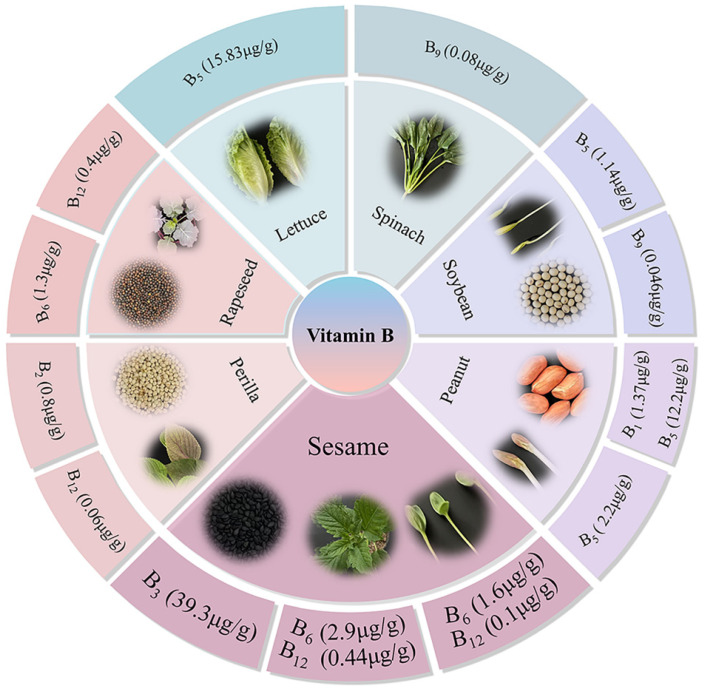
Diagrammatical illustration of significant B vitamin contents across the studied materials.

This study aims to characterize the interspecific and tissue-specific variations in B vitamin profiles across edible tissues of selected oilseed crops and leafy greens, as well as to assess vitamin B_3_ variations in sesame germplasms. Results revealed that significant interspecific differences in B vitamin variations across the five oilseed crop seeds and two leafy greens (Figure 6). Notably, sesame seeds exhibited exceptional vitamin B_3_ contents compared to other oilseeds crops, and this makes the crop regarded as a natural functional ingredient for niacin supplementation. Furthermore, sesame’s tissues such as leaves and seedlings are rich sources of vitamins B_6_ and B_12_. This multi-tissue potential of the crop allows for comprehensive dietary integration, from seed-based products to leafy greens and sprouted seedlings. Collectively, the unique B vitamin profile of sesame, along with genetic variability, offers the possibility of being an ideal candidate for a biofortification program—an approach that could contribute to efforts at addressing global B vitamin deficiencies and point to possible plant-based dietary solutions for the provision of antioxidant nutrients, based on the biochemical role of B vitamins in redox homeostasis. By leveraging the identified variations, future research should focus on vitamin bioavailability, processing effects, and genetic mechanisms underlying B vitamin accumulation to fully realize sesame’s potential as a functional crop. It is important to note that our tissue-specific comparisons were limited to the edible tissues for which viable samples were obtained, and they did not include all tissues of all six species. Future studies with comprehensive sampling of all species and tissues will further refine our understanding of B vitamin distribution across oilseed crops. And analytical limitations exist: the LC-MS/MS method used in this study cannot fully distinguish between biologically active B_12_ (cobalamin) and structurally similar inactive analogs (e.g., pseudocobalamin) commonly present in plants, potentially overestimating functionally available B12 levels [42].

## Figures and Tables

**Table 1:** Variation in vitamin B3 content among sesame germplasms.

Seed Coat Color	Number	Vitamin B3 (μg/g) Content				
		Max	Min	Mean	SD	CV
White	38	55.87	23.57	38.79	5.94	16.44%
Black	18	45.14	25.37	38.70	5.40	13.93%
Yellow	15	82.25	26.24	40.66	13.35	32.83%
Brown	21	48.64	24.08	37.90	7.18	18.94%
Total	92	82.25	23.57	39.28	7.86	15.12%

Min, minimum; Max, maximum; SD, standard deviation; CV, coefficient of variation.

## Data Availability

The original contributions presented in this study are included in the article. Further inquiries can be directed to the corresponding author.
